# An automated, high-throughput plant phenotyping system using machine learning-based plant segmentation and image analysis

**DOI:** 10.1371/journal.pone.0196615

**Published:** 2018-04-27

**Authors:** Unseok Lee, Sungyul Chang, Gian Anantrio Putra, Hyoungseok Kim, Dong Hwan Kim

**Affiliations:** 1 Convergence Research Center for Smart Farm Solution, Korea Institute of Science and Technology, Gangneung, Gangwon-do, South Korea; 2 Center for Intelligent & Interactive Robotics, Korea Institute of Science and Technology, Seoul, South Korea; Universidad Miguel Hernández de Elche, SPAIN

## Abstract

A high-throughput plant phenotyping system automatically observes and grows many plant samples. Many plant sample images are acquired by the system to determine the characteristics of the plants (populations). Stable image acquisition and processing is very important to accurately determine the characteristics. However, hardware for acquiring plant images rapidly and stably, while minimizing plant stress, is lacking. Moreover, most software cannot adequately handle large-scale plant imaging. To address these problems, we developed a new, automated, high-throughput plant phenotyping system using simple and robust hardware, and an automated plant-imaging-analysis pipeline consisting of machine-learning-based plant segmentation. Our hardware acquires images reliably and quickly and minimizes plant stress. Furthermore, the images are processed automatically. In particular, large-scale plant-image datasets can be segmented precisely using a classifier developed using a superpixel-based machine-learning algorithm (Random Forest), and variations in plant parameters (such as area) over time can be assessed using the segmented images. We performed comparative evaluations to identify an appropriate learning algorithm for our proposed system, and tested three robust learning algorithms. We developed not only an automatic analysis pipeline but also a convenient means of plant-growth analysis that provides a learning data interface and visualization of plant growth trends. Thus, our system allows end-users such as plant biologists to analyze plant growth via large-scale plant image data easily.

## Introduction

Plant phenotyping explores how the genome, interacting with the environment, affects observable plant traits (the phenome). These traits yield physical and biochemical information. Traditionally, such plant features have been identified simply by observing the appearance of plants or by destructive analyses. However, such methods are low-throughput, labor-intensive, costly, and time-consuming [1]. Therefore, interest is growing in image-based plant phenotyping using automated digital cameras [2, 3] to replace conventional manual observation. Several studies on advanced observation methods and plant growth analysis using two-dimensional images have been reported [4–9]. Such approaches offer continuity, consistency, objectivity and non-invasiveness, and reduce the effort, time, and cost required [10, 11]. When performing image-based plant phenotyping, it is important to classify the plants and control the image backgrounds. Robust classification facilitates accurate plant growth measurement and analysis. However, data extraction from images has several limitations. First, it is difficult to obtain consistent image data (i.e., the color distributions may be inconsistent because of shadowing). Also, soil and plant colors may vary over time. Second, image numbers may be low because the cameras are fixed (i.e., data diversity is lacking). Thus, images may differ significantly in terms of color distribution or be very noisy. It can therefore be difficult to distinguish the plant from the background; it is not easy to extract generalizable points in the presence of a lot of noise when only a few plant images are available.

To deal with these limitations, several plant-segmentation methods based on plant color have been proposed [12, 13]. However, serious errors are possible because segmentation performance is inadequate. Robust plant segmentation under various conditions remains challenging. To render plant phenotyping more continuous and accurate, not only image analysis but also environmental and watering data are required. However, studies to date have the disadvantages that the diversity of plant-image types is small, automatic data acquisition is not performed in real time, and sensors other than cameras are lacking. Therefore, in recent years, high-throughput phenotyping approaches have attracted interest; these use automated imaging systems not only in controlled indoor environments [14] but also in the field [15–19].

Robust, reliable, high-throughput phenotyping systems must not only collect growth data and morphological and physiological information but also perform step-by-step, traceable image analysis to assess biomass production and characterize gene function, among other possibilities [20, 21]. Various automatic, high-throughput phenotyping systems have been developed. The Phenoscope system (INRA, Versailles, France) screens 738 plants non-destructively [22]. LemnaTec (LemnaTec GmbH, Aachen, Germany) developed fully automatic greenhouse systems that monitor plants over time. Both of these systems allow fast and accurate analysis of plant growth; however, new problems have emerged. First, plant images are acquired by moving the plants to image-capturing chambers. Thus, the environmental conditions change when the plants are moved, and the plants may become stressed (e.g., by vibration or movement per se) [23]. Data acquisition may affect plant growth because the plants must be constantly moved. Second, high-throughput systems have “big data” problems that must be efficiently managed [24, 25]. In other words, it is necessary to track plant growth in real time and to manage and analyze large amounts of data efficiently. It is also challenging to remove noise automatically and analyze plant growth via preprocessing of massive data.

Here, we developed an automated, high-throughput, plant phenotyping system using hardware that captures images efficiently in real-time, together with machine-learning-based software. Our system differs from other systems in several ways. The hardware consists of a vision sensor with actuators, environmental sensors, and watering modules. This enables automated plant screening using a “sensor-to-plant” concept, unlike existing systems. The plants do not move; actuators with vision sensors move to acquire plant images in real time. Thus, plant stress is minimized even when images are acquired several times daily. Moreover, the actuator is free of vibration when moving. Therefore, it is possible to acquire stable large-scale plant images of good quality.

Our software pipeline consists of sequential-image-acquisition, pre-processing, plant-segmentation, and plant-growth-analysis stages. The pipeline allows automatic and accurate plant growth analysis using large-scale image data. When images are acquired, distortion is automatically corrected and the images are labeled and arranged. Moreover, imaged plants are automatically classified. A high-performance classifier is created rapidly using a machine-learning algorithm (Random Forest, RF). As superpixel color is used during training, the system is more robust to noise than a pixel-by-pixel approach; only plant images are acquired. Finally, plant growth is assessed by imaging defined regions precisely. The pipeline processes large-scale image data automatically and efficiently, extracts them as numerical data, and presents these in visual formats.

We show that our simple and robust hardware allows automatic, continuous and stable measurement of plant growth, together with the software pipeline that analyzes plant images robustly, commencing with image acquisition and processing, and proceeding to plant-growth analysis using a machine-learning method.

## Materials and methods

**Fig 1:**
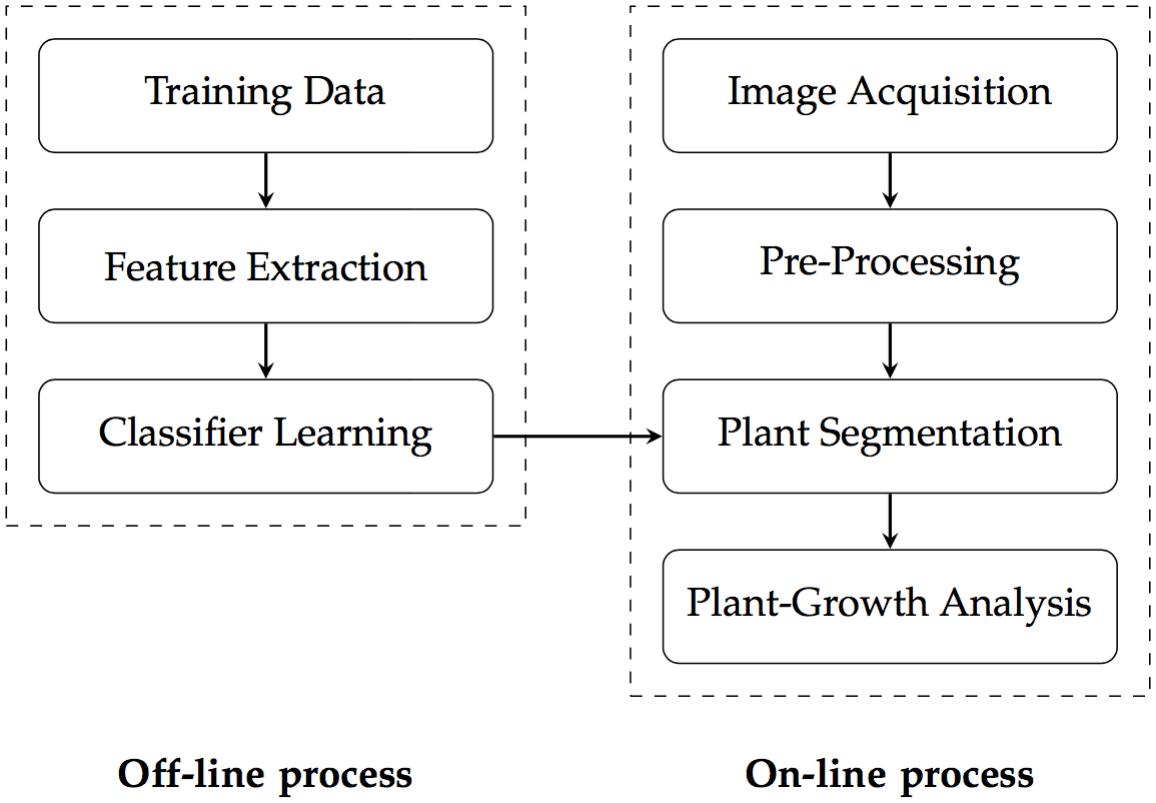
The pipeline of the plant phenotyping system.

The plant phenotyping system consists of both hardware and software. The hardware includes an image-capturing module, environmental-data sensors, and irrigation and light controllers. The imaging module employs an automatic robotic arm to acquire images of plant trays, and several sensors obtain environmental data. In addition, the frequency and amount of irrigation are controlled, and light intensity and duration are monitored. All data are stored and analyzed. The software includes five stages. First, the periodic images are assigned individual labels. Second, pre-processing consists of image warping and cropping. Next, classifiers are generated via an offline learning process and plants are segmented online using these classifiers (Fig 1). Superpixel-based feature extraction proceeds both online and offline. However, the system uses the features to generate classifiers offline and then classifies the plants online. The fourth stage is a post-processing stage; noise in the segmented images is removed and the large-scale data are processed. Finally, the system analyzes phenotypic information obtained over time.

Fig 1 summarizes the workflow.

### Hardware setup

**Fig 2:**
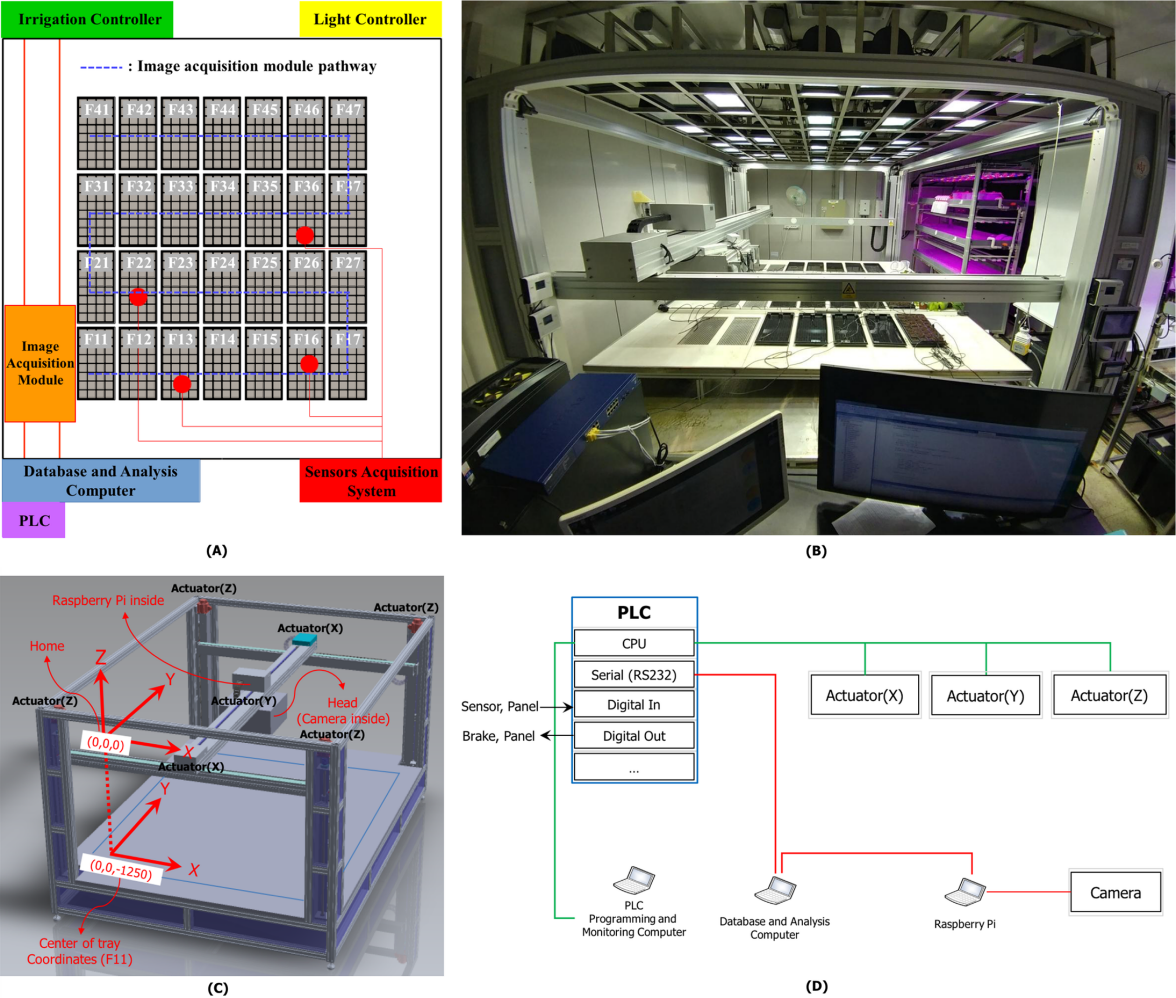
The hardware setup. A: System layout. B: The plant-phenotyping room. C,D: System configuration; the robotic arm moves the module from tray to tray and acquires top-view images.

In total, 28 plant trays are placed in a phenotyping room (Fig 2B) in a 4 × 7 matrix (Fig 2A); each tray is 52.5 cm × 26.5 cm in size and holds 32 6-cm × 6-cm pots in a 4 × 8-array. A single seed is planted in each pot. Unlike the existing phenotyping systems, which move plants via a conveyor belt to the phenotyping chamber, we use a moving module to acquire plant images. The image acquisition module (i.e., a moving module) moves over the pre-defined X, Y and Z coordinates of the plant trays, and consists of a head with a low-cost RGB camera (Logitech C920) and embedded computer (Raspberry Pi 3 Model B) as shown in Fig 2C. The module is operated by a programmable logic controller (PLC)-based system connected to a PLC programming and monitoring computer (Fig 2D); the PLC controls the actuators of the X, Y, Z axes to move the module to the pre-defined position, and sends a capture signal with a tray label. Sensors record temperature, relative humidity, soil moisture content, carbon dioxide (CO_2_) level, pH, electrical conductivity, photosynthetic photon flux density (PPFD), and irrigation and drainage rates. All data are stored on a database-and-analysis computer. The entire system occupies 4.3 m × 3.1 m in the phenotyping room.

### Image acquisition

Images are acquired by default every hour from 9:00 a.m. to 9:00 p.m. The default setting can be changed. In total, 364 images per tray are acquired daily; the system was tested over 90 continuous days yielding 32,760 images per tray. More than a million images were thus acquired (32 plants per tray). The process of image acquisition is as follows. The center of the image acquisition module head is parked in the “Home” coordinates (Fig 2C). The PLC controls the actuators to move the center of the module’s head to a specific plant tray’s relative coordinates such as (0,0,-1250). The module stops at those coordinates for about 10 seconds, and simultaneously sends a capture signal with a tray label to the database-and-analysis computer. The computer sends the signal to the module-embedded computer (i.e., Raspberry Pi) to acquire plant images (Fig 2C). The module-embedded computer is used to minimize the noise of the acquired images by minimizing the physical connection length of the camera’s USB cable. The module moves to another plant tray’s coordinates after finishing the acquisition. The module repeats these processes by moving along the pathway (Fig 2A). The module returns to “Home” coordinates at the end of the process. The cycle takes about 10 min. All images are stored in the embedded computer and automatically sent to the database-and-analysis computer after finishing one cycle. The images are stored based on the date and time of acquisition and arranged by plant and tray labels.

### Pre-processing

For accurate analysis, images should be acquired under the same conditions (e.g., exposure, focus, position, etc.). However, this can be difficult in real-life situations. For example, perspective distortion can occur if the camera is not exactly parallel to the plant or if the plant tray is slightly skewed. As the plant grows, the subject and the camera become closer; perspective distortion may develop. Such problems can compromise plant-growth measurements such as biomass estimation. We pre-process the data in three ways. First, image distortion is automatically corrected and preprocessed images are prepared for more accurate analyses. Second, each corrected tray image is cropped into images of individual pots; we thus track and analyze each plant individually. Finally, all images are managed and arranged by tray and pot labels, date, and time. Thus, large amounts of data are processed.

#### Correction of image distortion

**Fig 3:**
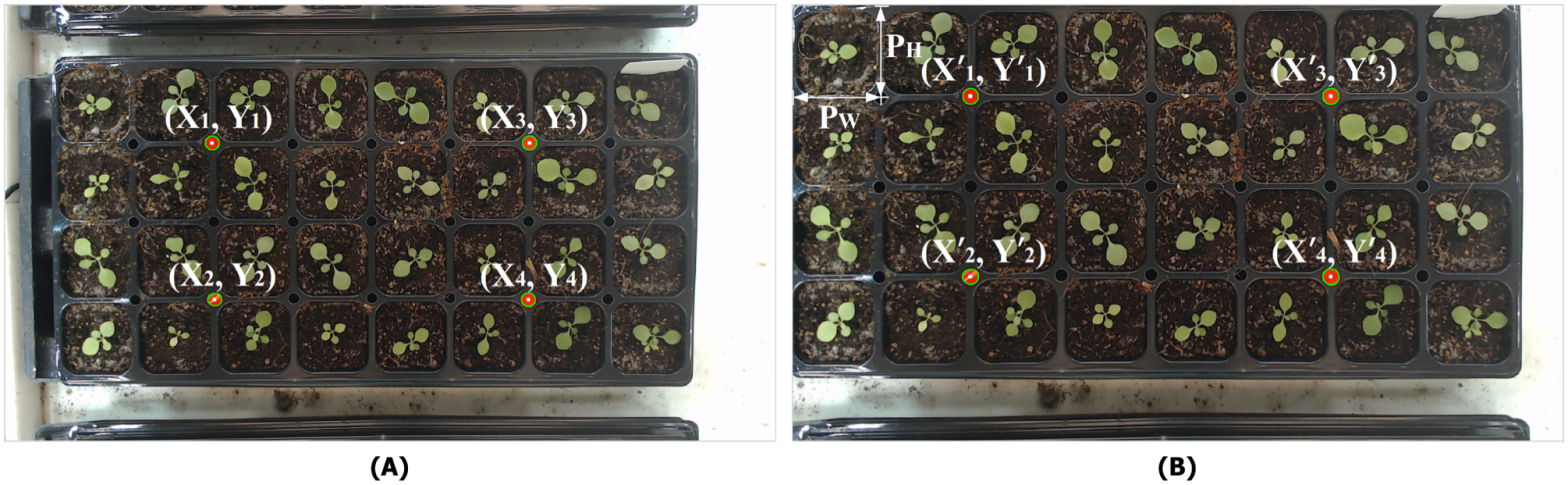
Top-view images. A: Top-view original tray images using color marker detection. B: The warped image based on the color markers.

Image distortion occurs when the sides of the tray are not parallel with the x and y axes of the image; we correct for this to allow accurate cropping of pots in the tray. In other words, we ensure that the plant area is not cropped incorrectly and that we analyze the correct area. Correction is based on the use of four color markers on each tray (Fig 3A). First, red markers are detected based on the color threshold, and then re-classified based on the binary threshold by converting RGB values to grayscale values. Contours are detected in the grayscale image, and those with areas less than the threshold (150 pixels) are removed. Adjacent contours are merged if the number of contours detected is over four and, finally, the four markers are found. If fewer than four markers are detected on a tray, the position of a missing marker is estimated by referring to its position on the previous or the next visit. The coordinates of the center points (i.e., the source coordinates) of the four markers are determined as shown in Fig 3A (e.g., *X*_1_, *Y*_1_). Then, the target coordinates (i.e., the coordinates of the corrected image) that are required for correction are calculated as follows. Let *P*_*W*_ be the width of a pot in a tray and *P*_*H*_ the height in the corrected image. *P*_*W*_ and *P*_*H*_ are pre-defined values that can be set directly to reflect the size of the acquired image, or determined automatically by the system. The coordinate of $X_{1}^{\prime }$ is 2*P*_*W*_ and that of $Y_{1}^{\prime }$ *P*_*H*_. Four target coordinates are obtained in the same way, and a homography matrix between the source and target coordinates is calculated. The source coordinates are perspective-warped to the target coordinates using this matrix. This step corrects and straightens distortion and the position of the tray. The final result is as shown in Fig 3B prior to accurate cropping.

#### Image cropping

**Fig 4:**
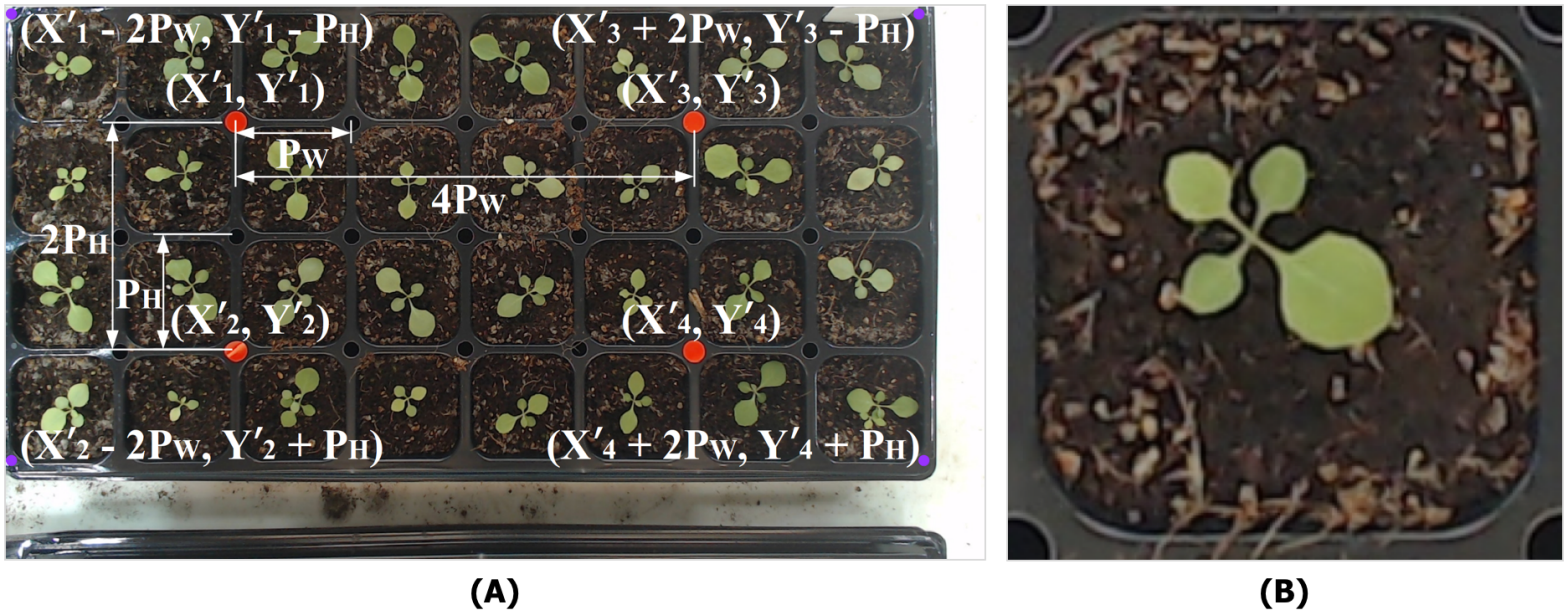
Top-view images. A: Estimation of tray edge coordinates. B: A cropped single-pot image.

Each plant sample is cropped accurately by reference to the tray edges and pot height and width. As the coordinates of the four markers and the pot size are shown in the corrected image, the system estimates the top-left/bottom-right tray coordinates. As shown in Fig 4A, the width between $X_{1}^{\prime }$ and $X_{3}^{\prime }$ is 4*P*_*W*_, and the height between $Y_{1}^{\prime }$ and $Y_{2}^{\prime }$ is 2*P*_*H*_. Therefore, the top-left edge coordinates are ($X_{1}^{\prime }-2P_{W}$, $Y_{1}^{\prime }-P_{H}$); Fig 4B illustrates cropping. We use commercial trays holding 32 plants. The camera obtains a tray image of 1,920 × 1,080 pixels, thus with pot images of 224 × 224 pixels (*P*_*W*_ × *P*_*H*_), and the images are subjected to warping and cropping. The pot size is adjustable. One cycle (28 images of a tray) yields 896 plant images.

#### Image arrangement

**Fig 5:**
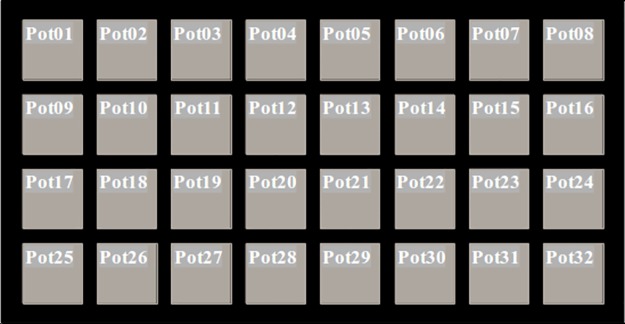
Top view of a tray showing pot labeling.

The system produces 11,648 cropped pot images daily, which are systematically organized and stored by tray and pot labels, date, and time of image acquisition. As shown in Fig 2A, the trays are labeled F11 to F47 on image acquisition and the pots Pot01 to Pot32 (Fig 5) on cropping. All storage is performed at the end of a cycle. For example, a pot image is saved as “F11_Pot01_date_time” in the F11_Pot01 folder. All images from germination to maturation for F11_Pot01 are saved in that folder. Thus, we obtain average plant growth/tray and growth data for all individual pots.

### Plant segmentation

Plant segmentation requires accurate foreground (plant) and background (soil, tray, moss, etc.) classification when measuring leaf areas, angles, and lengths. However, this can be difficult in practice because the background and plant may be similar in color and imaging and light conditions may vary. We use the following steps for robust image segmentation. Initially, preprocessing converts the color space from RGB to CIE L*a*b*. Each pixel is allocated to a superpixel group, and the color features of these groups are extracted and input to the trained color classifier, followed by binary classification. Then, the superpixel groups are re-broken into the original pixels to obtain the final image. Finally, noise is removed and plant area is rendered more accurately via post-processing. All of these steps are applied to all tray images. The processed values (e.g., plant area and length) and plant labels are converted into numerical data. Images in user-specified folders and subfolders can be subjected to image processing, again creating numerical data.

#### Feature extraction

**Fig 6:**
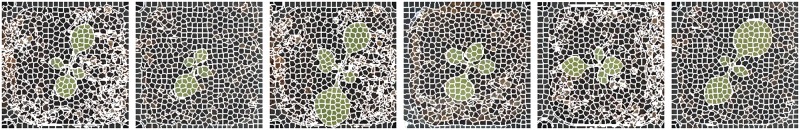
Superpixel images.

Common distinguishable features should be extracted to allow robust classification. The system creates generic features of plant areas only. The shape of a plant depends on the species, but all plants are similarly colored. Therefore, we use color as a key feature of classification. However, instead of simply using color per se, we group pixels with similar color characteristics. Such grouping reliably separates features from noise, via the following steps. Superpixel group creation is used to segment the image into groups of pixels that are approximately uniform in terms of color, size, and shape [26]. We use a Simple Linear Iterative Clustering (SLIC) algorithm to perform superpixel over-segmentation in the CIE L*a*b* color space. The SLIC method adapted a k-means clustering approach [27] to efficiently generate superpixels (Fig 6). When enhancing discrimination between the plant and the background, use of the CIE L*a*b* color space is appropriate, because it mirrors the human visual system, unlike the widely used RGB color space which employs 256-level quantization for each color, yielding more than 16 million different colors [28].

In summary, we transform plant images into the CIE L*a*b* color space and then apply the SLIC algorithm. The images are divided into *N* superpixels (*N* varies by the image), and the mean L, A and B values of each superpixel are calculated. Finally, an *N* (number of superpixels) × 3 (mean L, A and B) feature matrix is extracted as an image.

#### Segmentation

Plant-image segmentation seeks to extract only plant data from an image. Our system tracks changes in biomass over time by the individual pot, and uses these data in plant growth analysis. Plant segmentation is performed using features extracted from the images, as follows. An *N* × 3 feature matrix is created using the superpixel L*a*b* values; the value of *N* varies by image. This feature matrix is input to a trained color classifier generated using supervised learning datasets that are specially created for training employing the RF method. The classifier receives three superpixel features as inputs and returns a result of 0 or 1 for each superpixel; 0 is background and 1 foreground (the plant). The classified values should be applied to the original image (i.e., 224 × 224 pixels) as this is the image used to derive superpixel groups. For example, if an image contains 700 superpixels, 700 classifications will result. Each of the 700 superpixel groups can be eliminated or retained depending on the classification result (which contains information on the superpixel boundary). Finally, an image of the plant is obtained.

#### Post-processing

Post-processing consists of two steps, of which the first is image post-processing improving segmentation using images generated in the plant-segmentation step. Second, data post-processing generates numerical data from these images. During image post-processing, only the central plant area is required for growth analysis after the plant-segmentation step. However, segmented images may include noise. First, green background material such as moss may be segmented with the plant. Second, the area assigned to a pot may be invaded by leaves from other pots as growth proceeds. Image post-processing deals with these exceptional cases, allowing the required area to be delineated precisely. The procedure is as follows. We use erosion to eliminate noise caused by segmentation error. The system then creates groups of connected components to remove areas of plants from other pots. The probability is high that the plant of interest will be centered if the image is cropped correctly. Only the center plant area is retained; material connected to external components is removed. High-throughput image processing seeks to track changes in plant status continuously. When large numbers of continuously acquired images are obtained, automatic data post-processing is mandatory, as in our system.

During data post-processing, the plant area of each sample is transformed into numerical data over the entire time series and prepared for analysis. The system reads all subfolder images commencing with the root folders (i.e., the system default folders or user-defined folders) containing the various data, and then performs segmentation and image post-processing. Finally, the processed images are matched with their labels and values, and output to final files (e.g., .csv files).

### Plant-growth analysis

**Fig 7:**
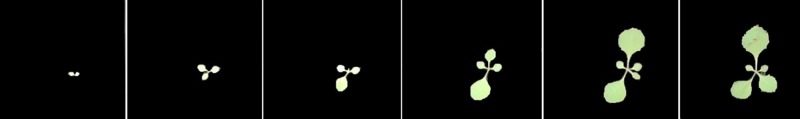
Plant-growth visualization; tracking plant area over time.

Biomass is an important aspect of plant phenomics. Biomass varies with the extent of stress and the levels of health-promoting materials. Our system analyzes biomass (plant area) via image- and data-processing, as follows. First, all plant data are processed from germination to harvest; we visualize morphological changes over time by reference to growth conditions (Fig 7). Second, numerical data (e.g., leaf area, length, etc.) are graphed, allowing evaluation of numerical changes rather than simply shapes in images.

## Results

In this section, we describe how we created the data used to train the classifier, and explain how we selected an optimal model by experimentally comparing learning algorithms. First, we describe our ground-truth image-creation interface for the data used in training, validating, and testing the classifier. Second, we explain how we designed classifiers using data created by the interface. In addition, we show how the system selected the optimal classifier. Third, we experimentally show how our classifier was trained using an appropriate learning algorithm. Finally, we visualized plant growth and area. The data and code are available at: https://github.com/ektf1130/high-throughput-plant-phenotyping-system.

### Ground-truth image-creation interface

**Fig 8:**
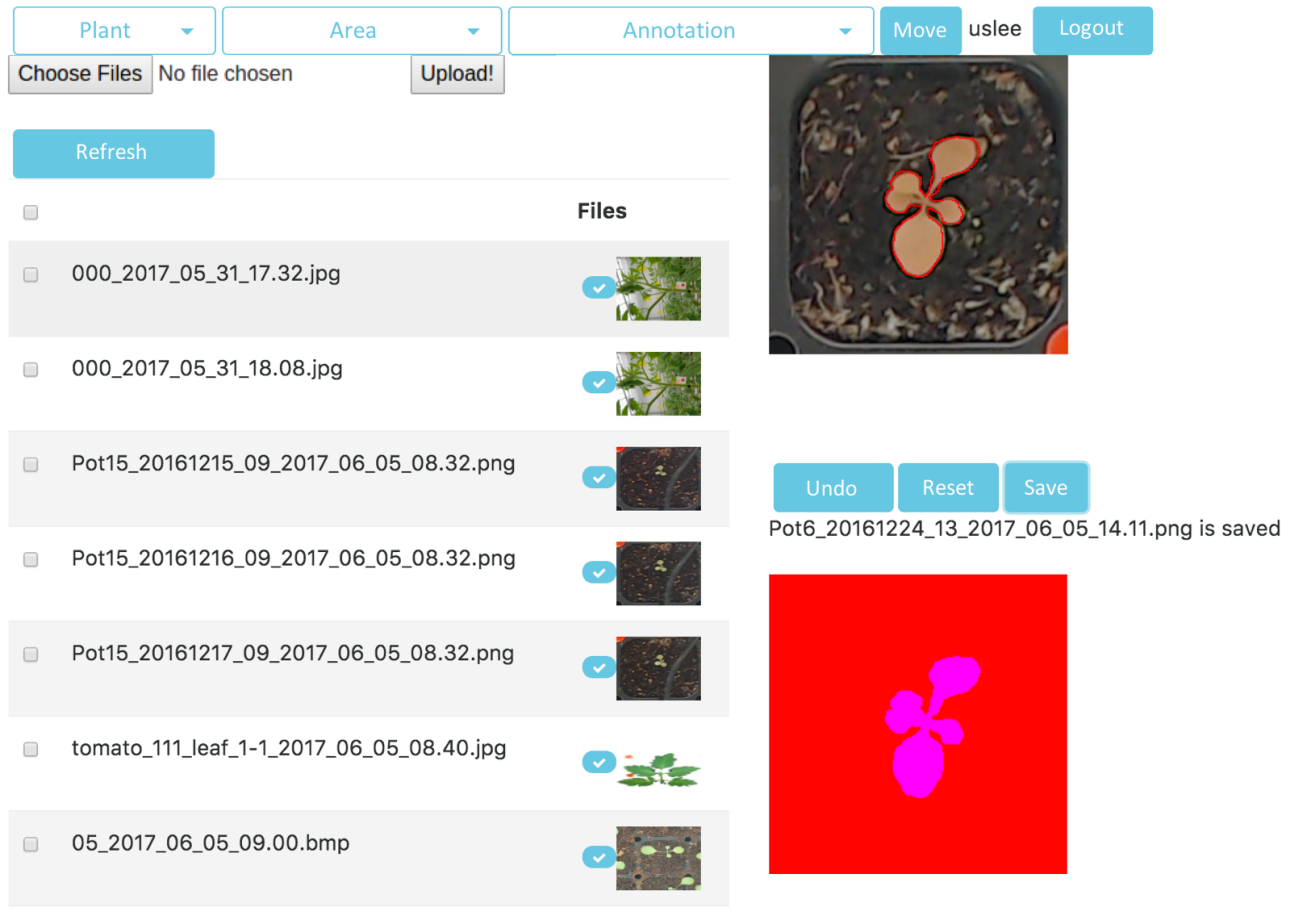
The ground-truth image-creation user interface.

Our system provides a ground-truth image-creation function. Generally, ground-truth plant imaging must consider many different conditions (e.g., light, plant size, etc.) when generating supervised, machine-learning-based, robust segmentation classifiers to cope with various natural environments. The requirements vary by the plant type. At least 100 ground-truth images are required for good plant segmentation, assuming that good-quality data are available. Image-editing tools such as Photoshop, GIMP, or open annotation tools have been used to create ground-truth images. However, the existing tools are inefficient when seeking to create many ground-truth images. In addition, the annotation tools are not suitable for creating segmentation classifiers; ground-truth areas are generally delineated by labeled rectangles. Therefore, we created a dedicated ground-truth image-creation algorithm allowing selection of multiple image objects using a drawing function (Fig 8) designed to read multiple images, process them simultaneously, and save them automatically.

Color value distributions vary by lighting conditions, the place where plants are grown, and the cultivation environment, reducing the accuracy of trained segmentation classifiers. We can use our interface to create additional ground-truth images as needed, thus improving classifier performance quickly and easily via re-learning.

### Classifier learning

**Fig 9:**
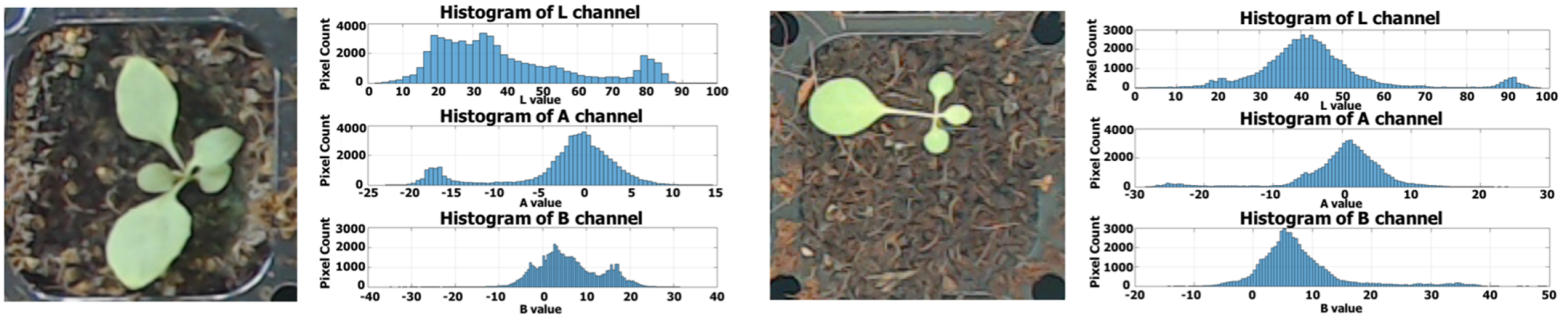
A validation dataset (left) and a test dataset (right).

Classifier learning proceeds offline (Fig 1). The classifier employs the RF algorithm running a training dataset created by the ground-truth interface. RF is an ensemble-learning algorithm featuring bagging [29]; we generate 100 binary classification decision trees (models) and choose the tree that optimizes the predicted values. Classifier performance varies because data are randomly selected during learning. However, RF is generally accepted as aiding in solving many classification problems. It is practical to re-teach the classifier if necessary; the learning time is short. We now explain how we generated a classifier trained using the extracted *N* × 3 features (i.e., the mean L*, a* and b* values of the superpixels) and *N* × 1 labeling (i.e., background 0; plant 1) using ground-truth images as input data. In total, 100 binary classification tree models were generated using the learning input data. Our training and validation ground-truth images for the classifier were created by manual operation of the creation interface. Images of all plant growth periods were input. In other words, differences in color distribution by plant size were considered. Also, the training data were chosen to reflect differences in color distribution caused by shadows from the module (i.e., the actuator), and illumination inhomogeneities on either side. We proceeded as follows when selecting the final model. The k-fold cross-validation method was used to generate classifiers; we created various training and validation datasets. On all images, 90% of the area was used for training and 10% for validation. Also, test datasets not used for either training or validation were employed to evaluate the classifier. These consisted of plant images obtained in environments that differed from those used in training and validation (i.e., we employed various cameras under different lighting conditions). The test datasets differed in terms of color distribution from the training/validation datasets (Fig 9). Finally, the system selected the final classifier and the hyperparameters using the validation results.

### Performance evaluation

We compared three types of classifier. The first classifier was trained using a Support Vector Machine (SVM) that found the maximum-margin hyperplane using support vectors [30]. The second classifier was trained using Multi-Layer Perception (MLP) (a feed-forward artificial neural network) [31]. The third classifier was trained using RF. We employed 843 ground-truth images when training and validating the classifiers and, finally, used images obtained in new environments to test the models. Each training and validation dataset was sampled using a k-fold cross validation method (k = 5). The test data differed in terms of color distribution from the training/validation data (Fig 9) and were used to test whether the final model worked well when images were acquired in other environments. We compared the F1-scores [32], precision-recall curves, and computation times using both validation and test data. Employing randomly generated data, we repeated these procedures 10 times to allow us to compare average classifier performances. The computer ran an Intel Core i7-4790K CPU operating at 4.00 GHz, and featured 32 GB of RAM and a 12-GB NVIDIA TITAN X video card (Pascal). The SVM employed Radial Basis Function (RBF) kernel, the MLP featured two hidden layers, each with 256 hidden nodes, and RF created 100 trees.

#### F1 scores

**Fig 10:**
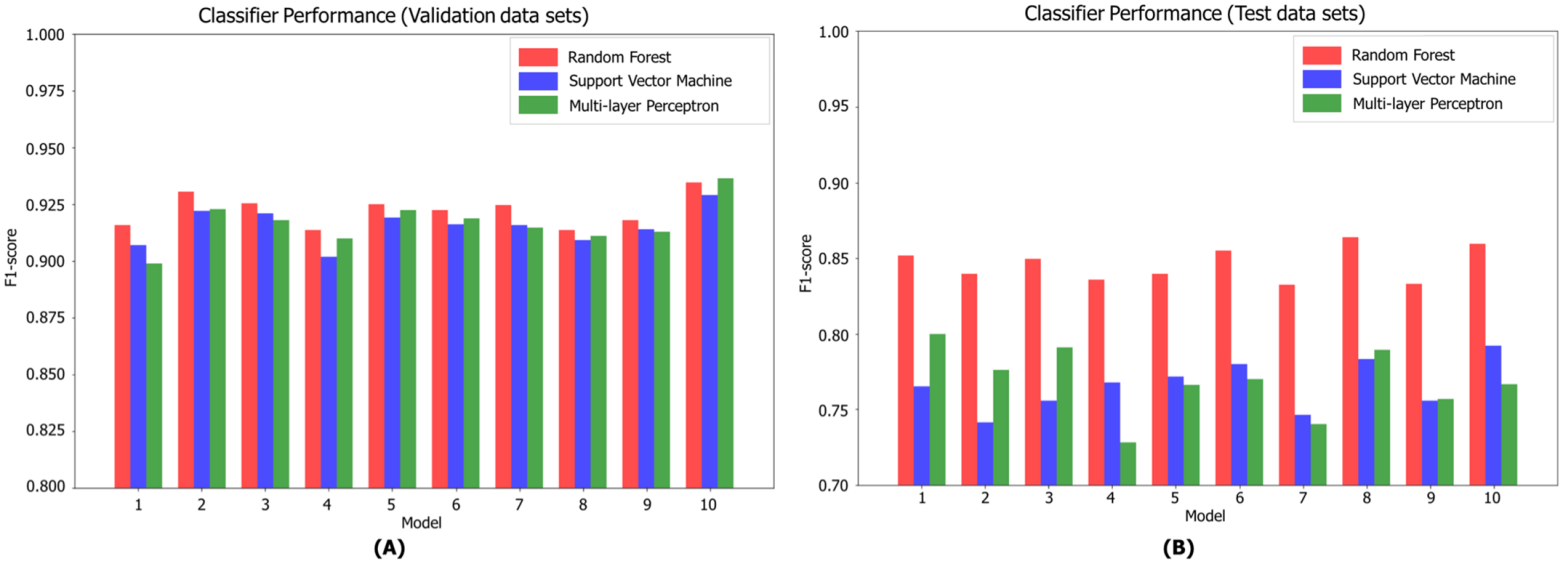
The F1 scores of the three classifiers. A: F1 scores of validation data sets. B: F1 scores of test data sets.

**Fig 11:**
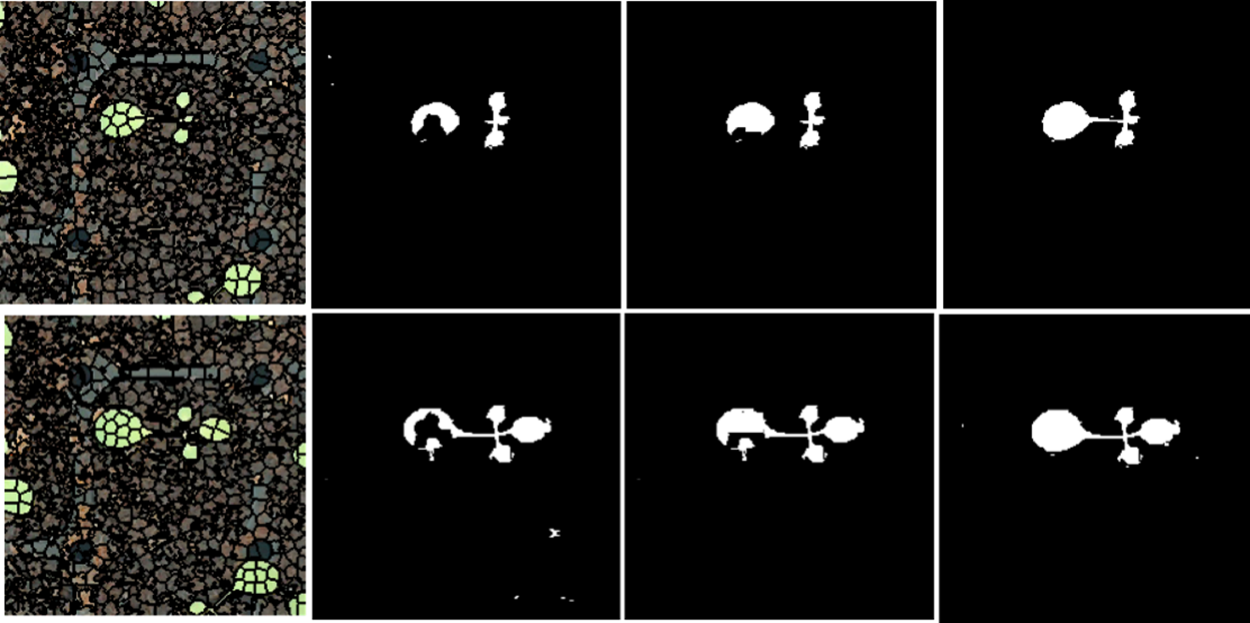
Segmentation results after post-processing using three trained classifiers (Original, SVM, MLP, and RF).

We used the F1-score to evaluate classifier performance. Accuracy could not be used to this end because classifier performance would be rated as over 70% accurate even if the classifier identified only background. Therefore, we evaluated the models by assessing precision and recall. We sampled 10 training and validation datasets using the k-fold cross-validation method. The model was trained using each training dataset, and the F1-score of each classifier was calculated using each validation dataset. The results are shown in Fig 10. Over 10 tests, RF yielded an average F1-score of 92.24%, and MLP and SVM scores of 91.67 and 91.56% respectively. Using the validation datasets, binary classification showed that all three classifiers performed well; later post-processing extracted more accurate plant areas. The F1-scores for the test datasets were lower (84.60, 76.90, and 76.60%, respectively). All three classifiers had lower F1-scores on the test datasets because the color distributions and L*a*b* color values varied. The results of segmentation of test datasets after post-processing are shown in Fig 11. RF performed best and, thus, we chose this training method.

#### Precision-recall curve

**Fig 12:**
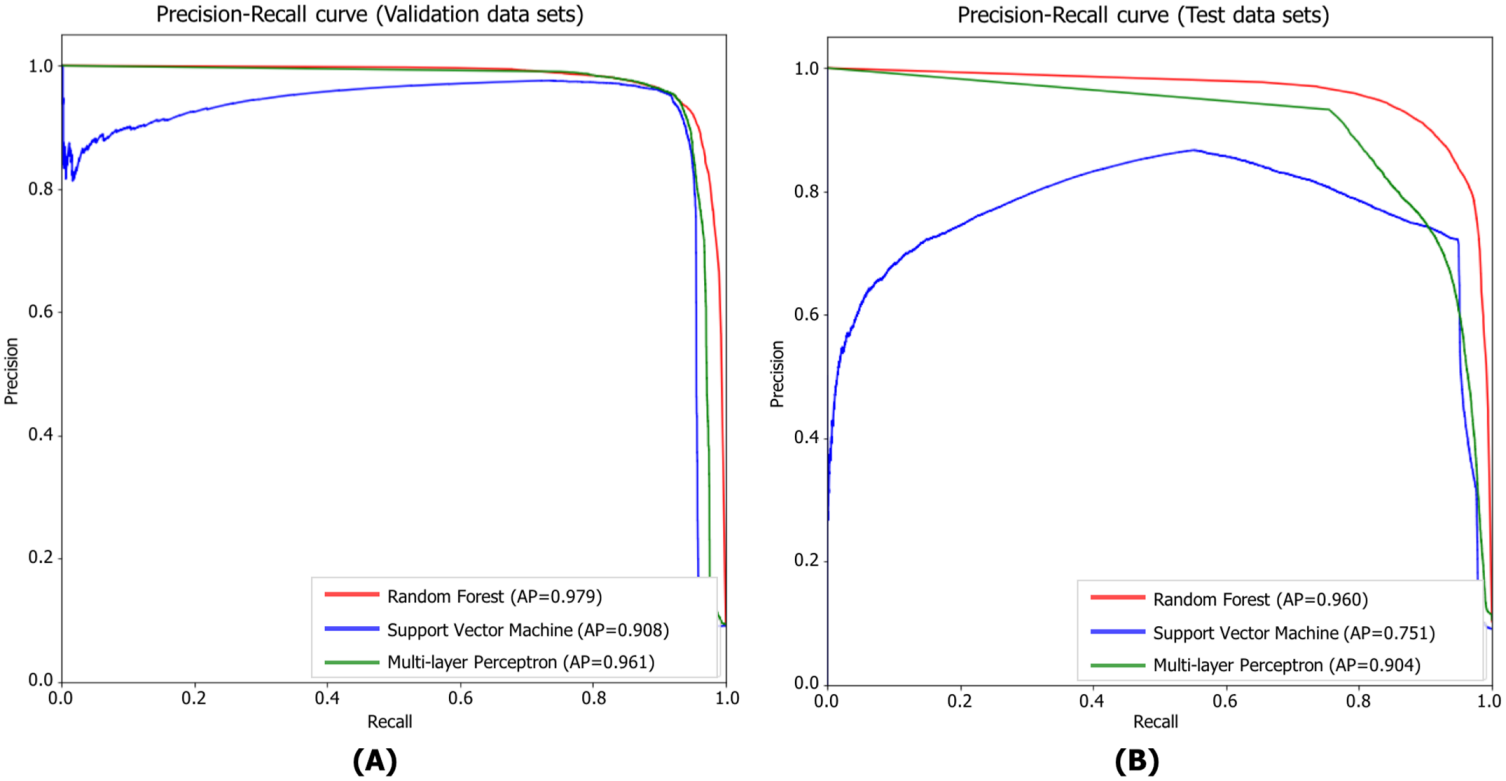
Precision-recall curves of the three classifiers. A: Precision-recall curves of validation data sets. B: Precision-recall curves of test data sets.

We used precision and recall as performance measures because the number of negative labels (i.e., background) exceeded the number of positive labels in our domains. Precision and recall can be used to create Precision-recall (PR) curves, which represent trade-offs between precision and recall. In general, PR curves clearly reveal performance differences between methods, more so than receiver operator characteristic curves [33]. We calculated the precision and recall values of each classifier using the highest F1-scores obtained over the 10 training iterations. The PR curves of the three classifiers were compared and evaluated using the validation and test datasets. As shown in Fig 12, RF yielded the highest Average Precision (AP) values of 0.979 (validation datasets) and 0.960 (test datasets). Furthermore, as shown in Table 1, RF also afforded the highest mean Average Precision (mAP) values; 0.9721 (validation data sets) and 0.9531 (test data sets). Thus, Random Forest was optimal.

**Table 1:** Comparison of mean Average Precision.

Learning method	Validation data sets	Test data sets
Random Forest	0.9721	0.9531
Support Vector Machine	0.8831	0.7296
Multi-layer Perceptron	0.9538	0.8443

#### Computation time

We compared the learning computation times of the three learning methods. The training data comprised 1.53 million × 3 matrices, and after data preprocessing such as superpixelization, the classifier took time to learn the data. For each of the 10 different training datasets, the average learning computational times were calculated. Each of the three learning algorithms of the scikit-learn [34] and tensorflow [35] libraries was used to this end. Most hyperparameters of the learning algorithms employed the library default values, albeit partially fine-tuned. The hyperparameters of Random Forest were *estimators = 100*, *criterion = entropy*, and the default parameters [34]. SVM used only the default parameters [34]. For MLP, the parameters were *2 hidden layer size = (256,256)*, *epoch = 10000*, *activation function = sigmoid*, *optimizer = AdamOptimizer (learning rate = 0.001)*, *dropout = 0.7 (learning time)*, and *1.0 (testing time)*.

The computation times are shown in Table 2. RF required about 20 s to train, SVM about 5 h, and MLP about 45 min (using the GPU). The learning time when RF was used was much less than those associated with MLP and SVM. Accordingly, RF is optimal when re-learning using new training data is required, and to define optimal classifier hyperparameters. Thus, RF not only offered optimal performance but also rapid learning.

**Table 2:** Comparison of learning computation time.

Learning method	Average of computation time (seconds)
Random Forest	19.6766
Support Vector Machine	18980.2678
Multi-layer Perceptron	2970.9253

### Visualization and trend analysis

**Fig 13:**
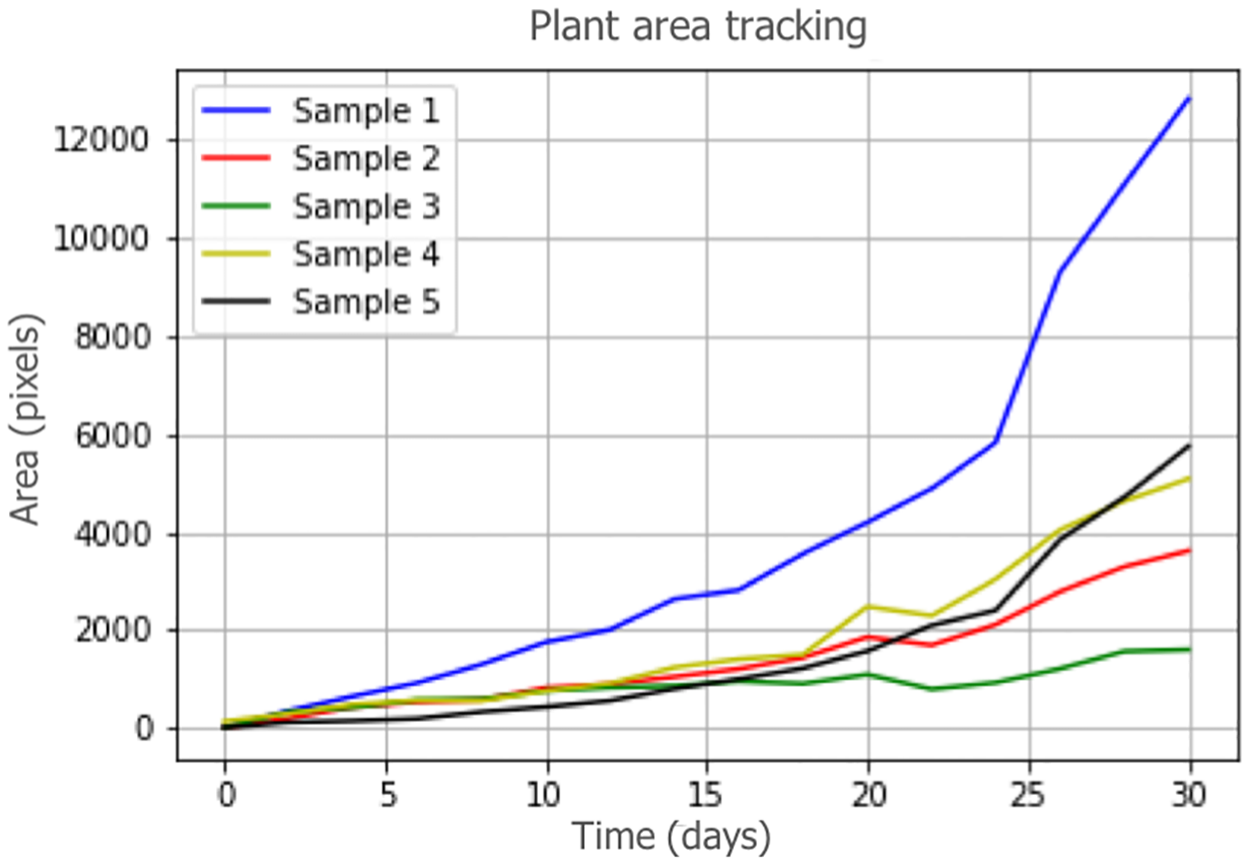
Plant-growth analysis; tracking plant area over time.

Changes in plant biomass can be visualized by sample or tray after plant segmentation and time-series data processing. Trends can be visually observed. We visualized the plant-area changes of five samples in one tray over 30 days (Fig 13). The plant growth rates varied. We can control plant growth by (for example) varying the irrigation of, and imposing nutrient deficiencies on, different samples in the same tray. The graph shows that Sample 1 had a greater biomass than other samples. The growth slope of Sample 1 is particularly steep in the interval 20-30 days. We can analyze environmental and irrigation controls imposed on Sample 1 to define factors significantly affecting growth. In addition, we can review environmental and irrigation information. Such data can be used to generate compound predictive models.

## Discussion

Our automated, high-throughput, plant phenotyping system differs from previous systems. Our system reduces stress by obviating plant movement; we employ the “sensor-to-plant” concept. Furthermore, we reliably obtain large-scale plant images of good quality. Unlike existing image-analysis pipelines [36], our pipeline consists of automatic pre-processing (i.e., distortion correction, image cropping) of large numbers of images employing simple reference markers, and images of the same tray or pot over time are labeled and mapped. Next, segmentation is performed, and relevant parameters (e.g., area, length, etc.) are extracted in .csv format and visualized. All processes are automatic, and it is easy to extract numerical from large-scale image data when sample-specific comparative analyses are required by end-users such as plant biologists.

Finally, we propose a robust classifier of noise; we employ superpixelization to this end. The boundaries of leaves and petioles are extracted without loss even if the image is noisy. Moreover, learning time is reduced by about 50% using superpixelization, because the number of pixels to be learned is reduced. Our classifier (created using RF) exhibited a good F1-score performance (about 92% on average) using only the color features of the superpixels, and also a short computational time. However, the F1-score performance degraded when new images differing in terms of color distribution were evaluated (Figs 9 and 10B). This was because the amount of water in the soil (i.e., reflecting differences in soil color) and light intensity were not uniform but, rather, depended on the position of the tray. In addition, color intensity changed when leaves overlapped. We will deal with these limitations by increasing the size of the training dataset to include images from all growth periods and all trays. We can create fresh training data using our ground-truth image-creation interface; re-learning is rapid when RF is the learning algorithm. Also, plant area estimation error using top-view plant images can occur because leaf movement is affected by the circadian rhythm [37]. Therefore, in future work, three-dimensional plant reconstruction using depth cameras or multi-view plant images will be performed to track leaf movements.

## Conclusions

In this study, we developed automated, high-throughput, plant image phenotyping hardware and a machine-learning-based pipeline to analyze large-scale image data; we observe plant growth consistently and precisely. The proposed hardware is robust and the analysis is convenient and practical; plant images are acquired rapidly and stably. Moreover, our system’s pipeline transforms huge amounts of real-time image data to numerical data and presents these data as graphs; plant growth can be readily explored. Furthermore, using superpixelization and RF, clean, precise, non-lossy images are obtained via plant segmentation, and the classifier can be re-learned rapidly if necessary. Our system can be used by plant biologists; it is easy to use and the data are automatically acquired and processed. Various comparative experiments will be possible using our system. For example, the effect of water stress can be examined by varying tray irrigation (i.e., to examine the relationship between plant growth and water stress). We are currently extending the pipeline to measure additional phenomic information such as the lengths, angles, and areas of individual leaves. It is expected that the system will be able to track plant growth, allowing detailed analysis and extended experiments (e.g., to examine the relationship between leaf angles and water stress).
